# Changes in Cuticle Components and Morphology of ‘Satsuma’ Mandarin (*Citrus unshiu*) during Ambient Storage and Their Potential Role on *Penicillium digitatum* Infection

**DOI:** 10.3390/molecules25020412

**Published:** 2020-01-19

**Authors:** Shenghua Ding, Jing Zhang, Lvzhu Yang, Xinyu Wang, Fuhua Fu, Rongrong Wang, Qun Zhang, Yang Shan

**Affiliations:** 1 Provincial Key Laboratory for Fruits and Vegetables Storage Processing and Quality Safety, Agricultural Product Processing Institute, Hunan Academy of Agricultural Sciences, Changsha 410125, China; shhding@hnu.edu.cn (S.D.); S152201609@hnu.edu.cn (J.Z.); ylzhu0115@163.com (L.Y.); wxy25994@163.com (X.W.); fhfu686@163.com (F.F.); zqun208@163.com (Q.Z.); 2 Longping Branch Graduate School, Hunan University, Changsha 410125, China; 3 College of Food Science and Technology, Hunan Agricultural University, Changsha 410128, China

**Keywords:** cuticle composition, wax morphology, cutin, ‘Satsuma’ mandarin fruit, postharvest storage, *Penicillium digitatum* infection

## Abstract

To elucidate the role of fruit cuticle in fungal infection, changes in cuticle composition and morphology of ‘Satsuma’ mandarin during ambient (at 25 °C) storage and their role in *Penicillium digitatum* infection were investigated. Results showed that the epicuticular wax yield increased from 1.11 μg cm^−2^ to 4.21 μg cm^−2^ during storage for 20 days and then decreased to 1.35 μg cm^−2^ as storage time prolonged to 40 days. Intracuticular wax content of fruits stored for 20 days showed a peak value that was 1.7-fold higher than that of fruits stored for 40 days. The contents of cutin monomers of fruits showed a decreased trend during storage, while their proportions in the cutin stayed stable. Acids were identified as the most abundant components in epicuticular wax independently of the storage time, followed by alkanes and terpenoids. Terpenoids were found as the predominant components in intracuticular wax during the whole storage, followed by alkanes and acids. The flattened platelets crystals of fruits at harvest changed into small granule-like wax ones after 10 days of storage then gradually distributed across the surface of the fruits as stored for 40 days. Results of in vitro tests showed that mycelial growth of *Penicillium digitatum* could be promoted by epicuticular wax and conidial germination could be inhibited by cutin at different storage stages. These results shed new light on the chemical basis for cuticle involvement in fungal infection.

## 1. Introduction

The plant cuticle is a lipidic layer synthesized by the epidermis, which surrounds aerial, nonlignified organs, including fruits. Plant cuticles are composed mainly by cutin, a polyester polymer matrix rich in hydroxylated and epoxy-hydrolated C_16_ and C_18_ fatty acids, embedded with amorphous waxes and a minor fraction of phenolics [1]. Plant cuticular waxes, mainly composed of very-long-chain fatty acids and their derivatives, can be divided into two wax layers: the intracuticular wax, embedded in the cutin polymer matrix, and the epicuticular wax on the outer surface of the cutin polymer matrix [2,3]. As the first barrier against the abiotic and biotic conditions in which they develop or are stored, the fruit cuticle limits the transpirational water loss, protects against physical, chemical, and biological attacks, as well as provides mechanical support to maintain plant organ integrity [3]. The self-assembling of plant epicuticular waxes during development introduces diverse three-dimensional crystal structures, such as massive crusts, granules, plates, platelet, filaments, rods, and tubules with a hollow center [2].

The fruit cuticle plays a decisive role in its development, being the first communication system with the surrounding biotic and abiotic environment, and is a modulator of postharvest quality [4]. In spite of this relevance, there are few studies focusing on cuticle modifications taking place during postharvest of fruit, which have often investigated the chemical compositions, crystal morphology, and key genes involved in formation for different cultivars and development. Sala et al. [5] reported that the epicuticular wax content of the ‘Satsuma’ mandarin fruits was lower than that of Navelina orange fruits, and alkanes, aldehydes, and fatty acids are the major wax constituents, whereas the primary alcohols and the triterpenoids are the minor ones. Wang et al. [6] also found that the epicuticular and total waxes of ‘Satsuma’ mandarin and ‘Newhall’ navel orange were mostly composed of aldehydes, alkanes, fatty acids, and primary alcohols. The altered ultrastructure and composition of cuticular wax from ‘glossy Newhall’ fruits lead to its glossy phenotype [7]. The formation of epicuticular wax crystals on the navel orange surface was shown to be dependent on the accumulation of high amounts of terpenoids while the underlying intracuticular wax layers have relatively low contents of aliphatic wax components but high loads of cyclic wax compounds [8]. Further transcriptome sequencing results showed that the decrease in most wax-related differentially expressed genes expressions in mutant fruit surfaces led to a reduced number of epicuticular crystals and final resulting in the glossy surface of mutant fruits [9]. Wang et al. [10] reported that cutin accumulation is synchronous with fruit expansion, while wax synthesis is synchronous with fruit maturation. However, little information focusing on the modification of ultrastructure and chemical compositions of ‘Satsuma’ mandarin fruit cuticle during postharvest is available. Meanwhile, changes in cuticle components, wax morphology, wax synthesis-related genes of many other fruits during postharvest has been well investigated, such as cherry fruit [1], apples [11], peach fruit [12], pear [13], and blueberry [14]. Several studies have also investigated the effects of postharvest treatments on the fruit cuticular components and wax crystal morphology during postharvest storage. Li et al. [15] found that ethephon increased cuticular wax density, accelerated wax crystal melting and fruit senescence during cold storage of ‘Starkrimson’ apple, while 1-methylcyclopropene (1-MCP) delayed these processes. Yan et al. [16] found that postharvest treatment of epigallocatechin-3-gallate (EGCG) can reduce accumulation of the fluid wax components and delay and lower the development of apple skin greasiness since the inhibitory effects of EGCG are to downregulate expression levels of genes involved in wax biosynthesis. Postharvest heat and CO_2_ shocks can also induce changes in cuticle composition and cuticle-related gene expression in ‘October Sun’ peach fruit [17]. In spite of being composed of similar chemical components, cultivar-related variations were observed in fruit cuticle composition and wax crystal morphology during postharvest storage. Hence, it is necessary to characterize the changes in fruit cuticle compositions and its contents, as well as the variations of wax crystal structure of citrus fruit during postharvest storage to master its biological function and explore a new strategy for maintaining fruit quality.

Fresh citrus fruits, from harvest to consumption, experience a period for transportation, storage, marketing, and delivery. During these periods, without an intact cuticle layer, fruits are more susceptible to biological diseases and physical damage, lowering its commercial value [18,19]. It is reported that cuticular waxes inhibited conidial germination of plant pathogens, such as *Podosphaera leucotricha* on certain varieties of apple [20]. Yin et al. [21] indicated that *n*-alkanes in low polar fraction and triterpenoids, fatty acid in polar fraction might have contributed to the antifungal properties of pear fruit cuticular waxes. Li et al. [22] observed that conidial germination and mycelial growth of *Alternaria alternata* could be inhibited by wax extracted from the pear fruit surface at different developmental stages. Furthermore, there have also been reports indicating the extracted cuticle waxes having a stimulatory effect on the germination and differentiation. *Blumeria graminis* f. sp. *Hordei* germination was more rapid and greater on the surfaces of intact than dewaxed barley [23]. Tang et al. [24] observed that the chemical composition and hydrophobicity of pear fruit cuticular wax are essential in facilitating fungal invasion by regulating the growth and differentiation of *Alternaria alternata* during the prepenetration phase. Several other ubiquitous very-long-chain aldehyde wax constituents are capable of effectively stimulating *Bluemeria graminis* prepenetration processes [25,26]. Variation in the cuticle composition may underlie differences in fruit resistance to microbial infection. The composition of the cuticle has been associated with the incidence and severity of fungal disease, particularly the presence of biologically active compounds that can reduce germination of conidia or inhibit germ tube growth of various fungi.

For citrus fruits, green mold, caused by *Penicillium digitatum*, is one of the main infectious diseases during postharvest storage. Previous reports have studied the infectious processes and mechanism of *Penicillium digitatum* for citrus fruits [27,28,29]. Despite the diversity of citrus cuticle composition and architecture being reported [5,6,7,8,10], information about the variations in the chemical composition of citrus fruit cuticle during storage, especially their potential roles in *Penicillium digitatum* infection, is not available. Similarly, there is a limited understanding of the relative importance and differing roles of waxes and cutin. ‘Satsuma’ mandarin is one of the main citrus cultivars in East Asia, while this cultivar shows poor storage ability. Therefore, the aim of this work was to study (1) the changes in components and morphology of ‘Satsuma’ mandarin fruit during ambient storage, and (2) the potential role of cuticle in *Penicillium digitatum* infection.

## 2. Results and Discussion

### 2.1. Postharvest Decay and Weight Loss

**Figure 1:**
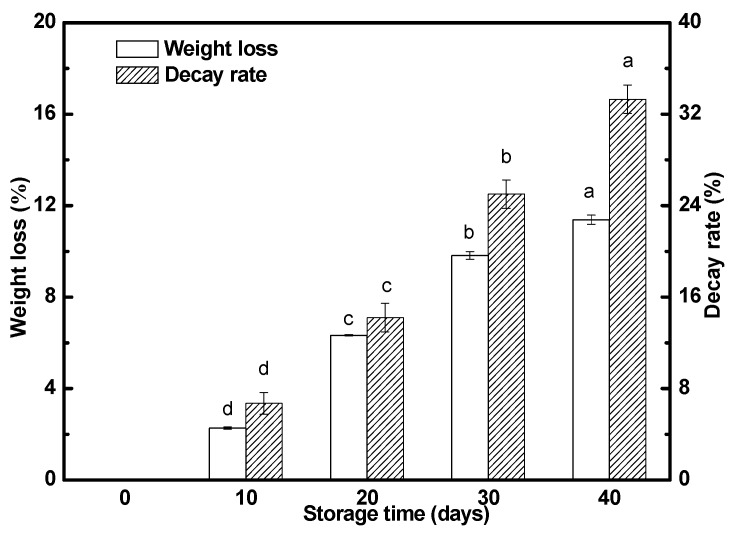
Postharvest weight loss and decay rate of the ‘Satsuma’ mandarin fruits stored at 25 °C with a relative humidity of 85–90% for 40 days. Data are means ± SD in three replicates. Columns for the same index marked by different letters are significantly different at *p* < 0.05.

The weight loss and decay rate of the ‘Satsuma’ mandarin fruits during storage were shown in Figure 1. Weight loss of the fruits stored at 25 °C increased significantly with the extension of storage time. Their weight loss was 2.3%, 6.3%, 9.8%, and 11.4% for 10, 20, 30, and 40 days of storage, respectively. The fruits stored at 25 °C began to rot after 10 days, and its decay rate reached 6.7%, 14.2%, 25.0%, and 33.3% for 10, 20, 30, and 40 days of storage, respectively.

### 2.2. Changes in Cuticular Fraction Contents of the Fruits during Storage

Changes in cuticle fraction contents of the ‘Satsuma’ mandarin fruits during storage were shown in Table 1. The epicuticular wax contents increased from 1.11 μg cm^−2^ to 4.21 μg cm^−2^ after 20 days of storage and then decreased to 1.35 μg cm^−2^ for 40 days of storage. In contrast, content of intracuticular wax decreased from 4.78 μg cm^−2^ to 3.70 μg cm^−2^ for 10 days of storage, then it also obtained the highest value (6.09 μg cm^−2^) for 20 days of storage, followed by a gradual decrease for 40 days of storage. It was found that the contents of the intracuticular wax were significantly higher than that of epicuticular wax as the fruits were stored for the same time. These results are in good agreement with previous findings in which intracuticular wax content of the sweet oranges was 5.4 times higher than that of epicuticular wax content at harvest time [30]. Total wax contents increased from 5.89 μg cm^−2^ to 10.20 μg cm^−2^ in the former 20 days, whereas it decreased to 5.01 μg cm^−2^ in the following 20 days of storage. These results were in good agreement with previous findings in which that wax yield of ‘Jesca’ peaches was found to be increased after 5 days at 20 °C [12]. Chu et al. [14] found that total wax content of ‘Brightwell’ blueberries decreased 17.9% after 30 days of storage at 4 °C. It suggested that the wax metabolism of postharvest fruits could be affected by cultivars and storage conditions. Li et al. [31] also found that ethephon accelerated apple fruit senescence, positively regulated total wax quantity and its alcohol, olefin, alkane, and acid constituents during cold storage, while 1-MCP inhibited these processes. The amounts of cutin ranged from 27.8 μg cm^−2^ to 52.5 μg cm^−2^, which were much higher than that of total wax in the same storage period. This observation agreed with the previous results for many other fruits, such as tomato [32], olive [33], and diospyros kaki fruit [34]. Cutin content decreased significantly from 52.5 μg cm^−2^ to 38.1 μg cm^−2^ in the former 10 days of storage then it kept stable in the following 10 days of storage, while it decreased to 28.2 μg cm^−2^ after 40 days of storage. Isaacaon et al. [35] also found that cutin deficiency in the tomato fruit cuticle consistently affected resistance to microbial infection and biomechanical properties but not transpirational water loss. L’Haridon et al. [36] reported that the cutin-deficient *Arabidopsis* mutants showed higher permeability and led to a release of more reactive oxygen species (ROS) to resist *B. cinerea.* Hence, the degradation of cutin during storage might induce a release of a series of ROS to improve the resistance to biological stresses, such as microbial infection, since the decay rate of fruits increased continuously during the ambient storage.

### 2.3. Changes in Epicuticular Wax Compositions during Storage

**Figure 2:**
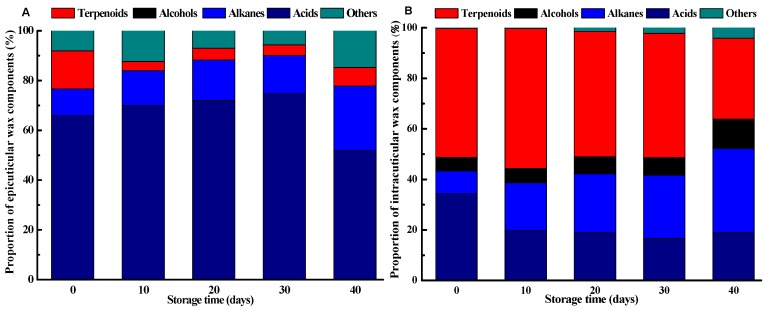
Epicuticular wax components proportion (**A**) and intracuticular wax components proportion (**B**) of the ‘Satsuma’ mandarin fruits during storage.

Changes in epicuticular wax compositions of the ‘Satsuma’ mandarin fruits during ambient storage were shown in Table 2. For the epicuticular wax fraction, 15 constituents were identified, including 9 kinds of acids, 4 kinds of alkanes, and 2 kinds of terpenoids (Table 2). Acids were identified as the most abundant components in epicuticular wax independently of the storage time, accounting for a proportion of 51.9–74.7% of the total epicuticular wax, followed by alkanes and terpenoids (Figure 2A). Similar results were also reported for the epicuticular wax components in the mature wild type ‘Newhall’ fruits, which were composed of fatty acids, *n*-alkanes, alcohols, and terpenoids [8].

For the *n*-fatty acids compositions of the epicuticular wax, they were comprised of a homologous series of even-numbered carbon atoms, except pentadecanoic acid. Among them, hexadecanoic acid, followed by eicosanoic acid and octadecanoic acid, was identified as the predominant fatty acid component in the epicuticular wax and its contents accounted for 30.4% to 54.8% of the total fatty acids content (Figure 2A). However, Wang et al. [6] found that hexacosanoic acid and octacosanoic acid were the main fatty acid components in the epicuticular wax of the ‘Newhall’ navel orange fruits, while octacosanoic acid and hexadecanoic acid were identified the dominating ones of the ‘Satsuma’ mandarin fruits. Liu et al. [8] also found that the acid constituents of the epicuticular wax in both wild and mutant oranges were influenced during the development and growth stages. Total contents of fatty acids in the epicuticular wax increased from 0.73 μg cm^−2^ to 3.03 μg cm^−2^ in the former 20 days, then decreased to 0.70 μg cm^−2^ on the 40 days as the samples were stored at 25 °C. Contents of the main long-chain fatty acids, hexadecanoic acid, eicosanoic acid, and octadecanoic acid also presented similar trends with that of total fatty acids contents during storage, respectively. For the fatty acid proportion to the total epicuticular wax, it increased gradually from 65.8% on the harvest day to 74.7% after 30 days of ambient storage, while it deceased sharply to 51.9% as the fruits were prolonged to 40 days of ambient storage (Figure 2A).

For the alkane components of the epicuticular wax, they consisted of homologous series of odd-numbered carbon atoms, including heptadecane, heptacosane, nonacosane, and hentriacontane. Heptadecane and hentriacontane were only detected in the epicuticular wax of fruits at harvest while both heptacosane and nonacosane were detected in the fruits throughout the whole storage (Table 2). Content of nonacosane increased 22.5 times in the former 20 days of storage, while it decreased by 44.4% in the rest 20 days of storage. A similar phenomenon was also observed for the change in the content of heptacosane during storage. It has been reported that alkanes content of 3 kinds of Asian pears increased after 7 months of storage at 3 °C [13], while it decreased significantly for the apples after 7 months of storage at 0 °C and this decline could be delayed with 1-MCP treatment [37]. For the alkane proportion to the total epicuticular wax, however, it showed an increased trend during the whole ambient storage, which increased from 10.8% on the harvest day to 25.9% after 40 days of storage (Figure 2A). Apart from fatty acids and alkanes, two terpenoids, namely squalene and friedelin, were also identified in the epicuticular wax. Squalene was only detected in the epicuticular wax of fruits at harvest. Content of friedelin of the epicuticular wax kept stable for the former 30 days of storage, while it decreased significantly on the 40 days compared with the harvest day (*p* < 0.05).

### 2.4. Changes in Intracuticular Wax Compositions during Storage

Intracuticular wax constituents of the fruits during ambient storage were shown in Table 3. In the intracuticular wax fraction, 28 constituents were identified, including 10 kinds of acids, 7 kinds of alkanes, 9 kinds of terpenoids, and 2 kinds of alcohols. Terpenoids were found as the predominant components in intracuticular wax during the whole storage, accounting for a proportion of 32.0–55.4% of the total intracuticular wax, followed by alkanes and acids (Figure 2B). A similar phenomenon was also reported for the leaves of *Ligustrum vulgare* [38] and tomato fruits [39]. It was showed that terpenoids were mainly restricted to the intracuticular wax in ‘Satsuma’ mandarin fruits at harvest, while acids and alkanes proportion of the epicuticular wax was higher than that of the intracuticular wax, respectively (Figure 2), which were the main distinction between epicuticular and intracuticular wax of the fruits [8,39]. Previous studies have developed several hypotheses focusing on the distribution of internal and external waxes of different components. For example, Buschhaus and Jetter [40] proved that the aliphatic substances showed a linear one-dimensional structure and the annular terpenoids presented a two-dimensional structure, which was incompatible in the agglomerated state. Therefore, terpenoids were filled in the cutin of polyester structure. The proportion of terpenoids, acids, and alkanes in the intracuticular wax showed different change trends during ambient storage. The alkane proportion of the intracuticular presented a gradual increase trend, which increased from 9.0% at harvest to 33.3% after 40 days of storage. However, the acid proportion of the intracuticular wax decreased from 34.3% to 19.7% during the former 10 days of storage and it did not change significantly during the following 30 days of storage. For terpenoids proportion, it increased from 51.0% to 55.4% after 10 days of storage, while it decreased drastically from 49.2% to 32.0% during the later 10 days of storage (Figure 2B).

Changes in intracuticular wax components of ‘Satsuma’ mandarin fruits during ambient storage were shown in Table 3. Though both of the intracuticular and epicuticular wax were composed of acids, alkanes, and terpenoids, their compositions and related contents were strikingly different. For the *n*-fatty acids compositions of the intracuticular wax, they were also mainly comprised of a homologous series of even-numbered carbon atoms, except pentadecanoic acid and heptadecanoic acid. These two acids were only detected in the fruits at harvest. Among the acids, hexadecanoic acid showed the highest content, followed by octadecanoic acid and oleic acid. Its content presented a gradual decrease trend during storage except for the 10 days of storage, and a similar phenomenon was also observed for the total acid content. Terpenoids showed the highest contents in the intracuticular wax during the whole storage period. It mainly included friedelin, *β*-amyrin, lup-20(29)-en-3-one, and squalene. Among them, friedelin was identified as the component with the highest content of the intracuticular wax, while it was not detected in the epicuticular wax in the whole storage. Terpenoid contents obtained the highest value (4.35 μg cm^−2^) after 20 days of storage then decreased significantly to 2.27 μg cm^−2^ during the following 20 days of storage (*p* < 0.05). A similar change trend was also observed for the friedelin content. In agreement with previous reports, the current study further confirmed previous studies that the fruit epicuticular waxes consisted predominantly of aliphatic compounds, and a similar pattern of aliphatics was presented in the intracuticular wax compartment but was mixed with a large number of cyclics [6,7,8]. It was previously proved that the major portion of the transpiration barrier was located in the intracuticular wax layer and was largely determined by the aliphatic constituents since an increase in the level of cuticular terpenoids could not compensate for the loss of aliphatics [41]. Previous studies showed that the enhanced triterpenoids would impair the transpiration barrier function of cuticles due to the increase of amorphous waxes [35,42].

### 2.5. Changes in Cutin Monomers during Storage

Cutin monomer compositions and their contents of the ‘Satsuma’ mandarin fruits during ambient storage were shown in Table 4. Nine cutin monomers were identified in the cutin fraction at harvest, namely, cinnamic acid, pentadecanoic acid, hexadecanoic acid, hexadecanedioic acid, octadecanoic acid, octadecanedioic acid, tetracosanoic acid, octacosanoic acid, and phenol. Wang et al. [10] also identified cinnamic acid in ‘Newhall’ fruits during development, while they detected 10-oxo, 16-hydroxyhexadecanoic acid, 10,16-dihydroxyhexadecanoic acid, 16-hydroxyhexadecanoic acid, and ferulic acid. Baker and Procopiou [43] also reported that the mandarin fruit cutin contained 1% of hexadecanoic acid, and three other acids, namely, 16-hydroxyhexadecanoic acid, 16-hydroxyoxohexadecanoic acid, and dihydroxyhexadecanoic acid. These differences could be ascribed to the diverse cultivars, developmental stages, or the origin resources. Octadecanoic acid showed the highest amount among the detected cutin monomers in the whole storage periods, ranging from 9.17 μg cm^−2^ to 17.02 μg cm^−2^, followed by cinnamic acid, with the range from 6.26 μg cm^−2^ to 13.91 μg cm^−2^. The amount of these two cutin monomers showed a decreased trend during ambient storage. A similar phenomenon was also observed for pentadecanoic acid and octadecanedioic acid. Previous study also showed that content of cutin monomers started to decrease during storage in ‘Newhall’ fruit peel [44], which suggested that cutin might be degraded by lipase.

**Figure 3:**
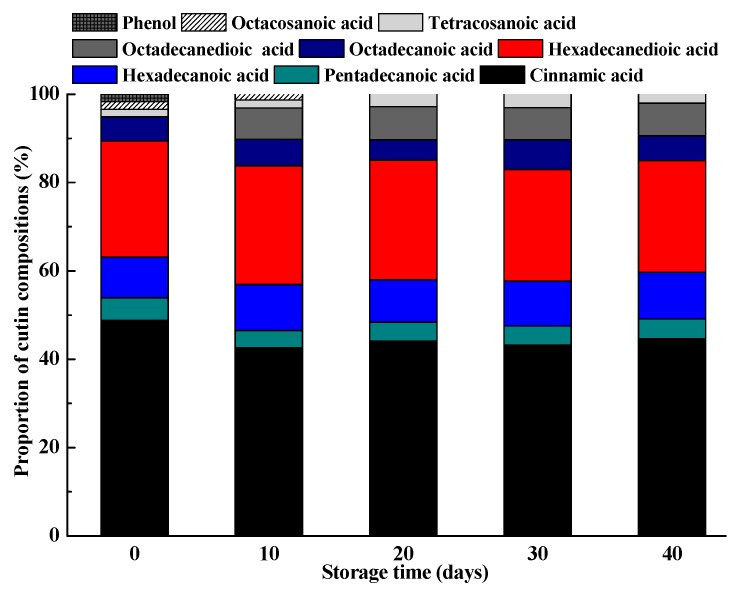
Cutin monomers composition proportion of the ‘Satsuma’ mandarin fruits during storage.

Although these cutin monomers decreased gradually during fruit storage, their proportions in the cutin kept stable especially in the later storage stages (Figure 3). The regression analysis showed that there was a negative association between weight loss and the amount of octadecanoic acid or cinnamic acid, with *r* values of −0.90 and −0.96, respectively. Pararsons et al. [45] also reported that total cutin monomers or certain monomers of cutin, including *ω*-hydroxyhexadecanoic acid and 16-dihydroxyhexadecanoic acid, showed negative associations with water loss, respectively. Changes in the cutin monomers and their proportions during storage could affect the functions of the fruit cuticle, which would further influence the quality attributes and shelflife of the postharvest fruits. Marga et al. [46] found that the ratio of hexadecanoic acid to octadecanoic acid for cutin monomers affected the mechanical properties of the cuticles of *Cirsium horridulu*, which showed an elastic state as the hexadecanoic acid presented a high proportion in the cutin monomers, while exhibited a rigid state as the amount of hexadecanoic acid and octadecanoic acid was approximately equaled.

### 2.6. Changes in Crystal Structure of Epicuticular Wax during Storage

**Figure 4:**
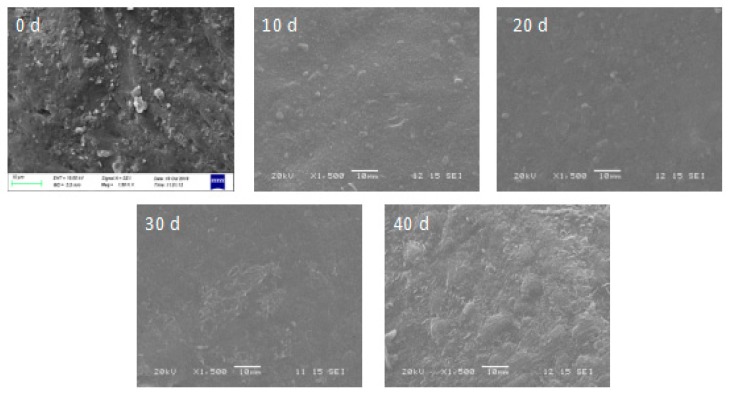
Epicuticular wax crystal structure of the ‘Satsuma’ mandarin fruits during storage for 40 days was detected at ×1500 magnification.

Scanning electron microscopy (SEM) observation revealed that the epicuticular wax crystals of ‘Satsuma’ mandarin at harvest are distributed with flattened platelets (Figure 4), which are also observed in mature citrus fruits [6,8,47]. The flattened platelets crystals of fruits stored at harvest changed into small granule-like wax ones after 10 days then gradually distributed across the surface of the fruit as the storage time was extended to 40 days. Crystal structure diversity was affected by temperature and this is in accordance with the observation of cactus pear fruit by López-Castañeda et al. [48], which found that heat treatment caused rearrangement of the epicuticular wax layers, minimizing or eliminating cracking. Simultaneously, the altering of epicuticular components was another important reason for crystal structure changes. Epicuticular wax can provide a physical barrier for microbial infection and altered physical fruit surface structures may provide significant cue(s) for the initiation and execution of pathogen [23,47]. The wax melting during storage leads to cracks on the surface of the fruit and increase the probability of microbial infection.

### 2.7. Effect of Different Cuticle Extracts on Mycelium Growth and Conidial Germination of Penicillium digitatum In Vitro

**Figure 5:**
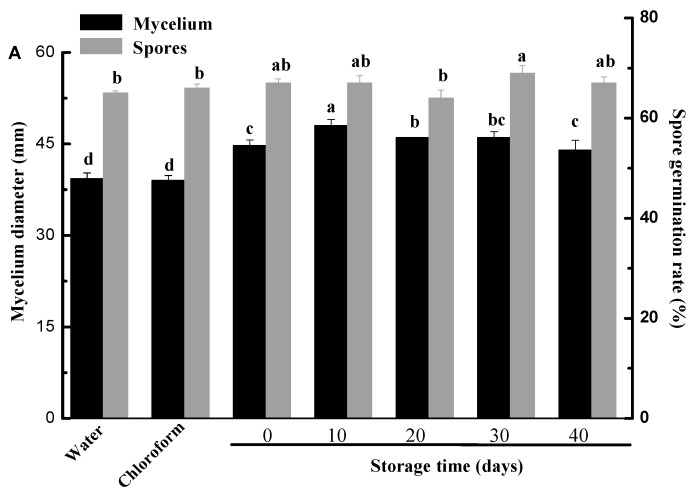

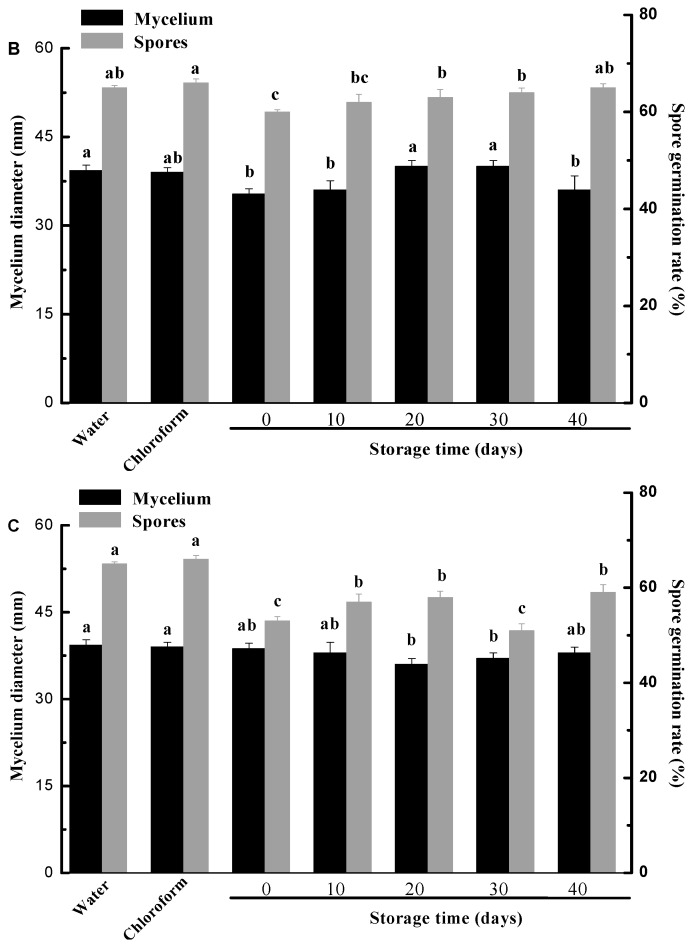
Effect of different cuticle fractions on mycelial growth and spore germination of *Penicillium digitatum* in vitro (**A**) Epicuticular wax extracts; (**B**) Intracuticular wax extracts; (**C**) Cutin extracts; Data are means ± SD in three replicates. Columns for the same index marked by different letters are significantly different at *p* < 0.05.

The effects of different cuticle extracts isolated from fruits during storage on mycelium growth and spore germination of *Penicillium digitatum* were presented in Figure 5. The epicuticular wax showed significant promotion to the mycelium growth of *Penicillium digitatum* compared with controls as showed in Figure 5A (*p* < 0.05). However, the intracuticular wax presented different affections on the mycelium growth of *Penicillium digitatum* compared with controls (Figure 5B). Intracuticular wax from the fruits stored for 20 days or 30 days did not significantly affect the mycelial growth (*p* > 0.05), while the one from the rest storage periods inhibited significantly (*p* < 0.05) compared with the controls. The different effects between epicuticular and intracuticular wax on the *Penicillium digitatum* could be ascribed to the distribution of terpenoids, which were showed antibiotic activity and mainly restricted in the intracuticular wax. Yin et al. [21] reported that terpenoids and alkanes from Asian pear fruit cuticle inhibited the germination of *Alternaria* spores and the growth of its mycelia. In this study, terpenoid contents of the intracuticular wax were 14.9–22.7 times of the epicuticular wax during ambient storage and alkanes presented similar distributions. Naziri et al. [49] also found that enhanced squalene produced by wild-type *Saccharomyces cerevisiae* strains eliminated the ROS and postponed the decline and fall of fruits. Liu et al. [50] reported that farnesol induces apoptosis and oxidative stress in the fungal pathogen *Penicillium expansum.* For cutin, there were no significant differences for the mycelial growth among four different storage periods (*p* > 0.05) (Figure 5C). Although no significant difference was found between epicuticular and intracuticular wax effects on spore germination, a marked inhibition of cutin on spore germination was recorded. Chloroform solvents alone had no significant effect on mycelial growth and spore germination. Plant cuticle has the role of prevention or facilitation of fungal invasion in the process of infection [51]. The facilitation role of epicuticular wax was confirmed by this experiment, although the promotion rate varied with different storage stages. This result showed that there were some promoting compounds in the epicuticular wax of citrus fruits. Hegde and Kolattukudy [52] found that long-chain fatty acids stimulate development of *Magnaporthe grisea* infection structures. In this study, fatty acids were shown to be major components of epicuticular wax. Tang et al. [24] also reported that high surface hydrophobicity resulting from cuticular wax of pear also stimulated infection structure formation of *Alternaria alternata*.

## 3. Materials and Methods

### 3.1. Experimental Materials and Pathogen

Mature citrus of ‘Satsuma’ mandarin (*Citrus unshiu*) was harvested on 10 October 2018 in Shimen County, Hunan Province, China. After harvest, the fruits were delivered immediately to the laboratory. Fruits with uniformity of size and color, free from visible blemishes, disease, and/or physical damage, were selected. The fruits were stored in several incubators (LHS-250HC-II, Shanghai Yiheng Scientific Instrument Co., Ltd., Shanghai, China) at 25 °C for 40 days with a relative humidity of 85–90%. A total of 30 fruits were withdrawn every 10 days for cuticle analysis. The decay in each replication of 50 fruits was examined visually, counted every 10 days and expressed as percentage of rotting fruit.

*Penicillium digitatum* was cultured on potato dextrose agar (PDA) after purchased from Microbial Culture Collection, Guangdong province, China (www.gimcc.net). Spores were removed from 7-day-old PDA cultures and suspended in sterile distilled water containing 0.05% (*v*/*v*) Tween 80. The suspension was filtered through two layers of sterile cheesecloth to remove any adhering mycelium. *Penicillium digitatum* spores were adjusted with sterile water and counted with a hemocytometer, respectively.

### 3.2. Determination of Pericarp Surface Area

Fruit surface area was assessed by using the method reported by Parsons et al. [45] with some modifications. Citrus fruit peel was weighed and forty discs (1.7 cm in diameter) were punched out, and the surface area was calculated (Equation (1)).

(1) $$\mathrm{Fruit}\;\mathrm{surface}\;\mathrm{area}\;({\mathrm{cm}}^{2})\;=\;\frac{X\;\times \;D}{Y}$$

where *X* is the weight of the fruit peel (g), *D* is the area of the forty discs (cm^2^), and *Y* is the weight of the forty 1.7 cm discs (g). Three replicates of each 10 fruits were used for determination of pericarp surface area.

### 3.3. Cuticle Extraction and Analysis

#### 3.3.1. Epicuticular and Intracuticular Wax Extraction

Cuticular wax extraction was performed as described by previous reports [6,8]. Briefly, commercial gum arabic powder was used with chloroform to remove any soluble lipids and residues. Once dissolved in distilled water (1 g mL^−1^), the glue solution for epicuticular wax extraction was applied to the entire fruit surface using a small paintbrush and air-dried for 3–5 h until a dried and stable polymer film formed. Then, the polymer films were collected, dissolved in 30 mL of distilled water, and extracted with chloroform at room temperature, and the process was repeated twice. After the adhesive treatments, the intracuticular wax was extracted by immersing the same fruit twice in CHCl_3_ for 30 s. A volume of 200 µL (1 µg µL^−1^) *n*-tetracosane was added as an internal standard and dried completely under nitrogen gas (N_2_). The dried waxes were stored at −20 °C before gas chromatography-mass spectrometry (GC-MS) analysis. Three replicates of 10 fruits each were used for epicuticular and intracuticular wax extraction.

#### 3.3.2. Epicuticular and Intracuticular Wax Analysis

Wax analysis was carried out according to the procedure as described by Parsons et al. [45] with some modifications. Briefly, extracts were incubated in pyridine for 30 min at 50 °C, followed by a 40 min of derivation treatment at 60 °C using bis-*N*,*N*-(trimethylsilyl)-trifluoroacetamide (BSTFA, Sigma, Darmstadt, Germany). After the evaporation of excessive BSTFA under N_2_, the derivatives were redissolved with chloroform for GC-MS analysis. The wax mixtures were conducted on capillary GC (Agilent 7890A with an HP-5 MS column 30 m, 0.25 mm, 0.25 μm) with He carrier gas inlet pressure programmed for a constant flow of 2 mL min^−1^ and a mass spectrometric detector (Agilent 5975C, 70 eV, *m*/*z* 50–600). GC was carried out with temperature-programmed automatic injection at 70 °C and an oven temperature for 1 min at 70 °C, then raised by 20 °C min^−1^ to 200 °C, held for 2 min at 200 °C, raised by 5 °C min^−1^ to 300 °C and held for 20 min at 300 °C. The individual wax components were identified by a comparison of their mass spectra with those of authentic standards and literature data. Quantitative determination of wax components was done by comparing their GC peak areas to that of the internal standard. The amount of cuticular wax components were normalized to these surfaces areas.

### 3.4. Cutin Isolation and Its Monomers Analysis

After extraction of cuticular wax, the fruits were then peeled for cutin isolation and analysis of cutin monomers according to the procedures of Wang et al. [10] with some modifications. To avoid any pollution, the enzyme solution was changed every 2 days until the discs had little or no cellular debris attached to them. The enzymatic cuticle membranes were immersed with methanol for 6 h, then were dewaxed with dichloromethane for 17 h, followed by tetrahydrofuran for 20 h. Dewaxed cuticular membranes were depolymerized in 3 mL of 14% (*v*/*v*) of boron fluoride methanol solution at 70 °C for 16 h. After cooling, 2 mL saturated NaHCO_3_ solution was added to the methanolysate. The cutin monomers were extracted three times with 2 mL chloroform for 10 min, followed by thorough mixing and centrifugation at 20 °C. Finally, the extracts evaporated in a stream of N_2_. Dried cutin monomers were derivatized with BSTFA in pyridine for 40 min at 90 °C, and 200 μL (1 μg μL^−1^) of *n*-tetracosane were also added as an internal standard.

After the evaporation of pyridine and excessive BSTFA under N_2_, the derivatized samples were redissolved in chloroform for GC-MS analysis. The composition of the cutin monomers was analyzed by the same capillary GC. The oven temperature was programmed as 2 min at 50 °C, followed by a 10 °C min^−1^ to 150 °C, held at 150 °C for 1 min, increased by 3 °C min^−1^ to 290 °C, and held at 290 °C for 20 min. Cutin monomers were identified and quantified by the same method described above in epicuticular and intracuticular wax analysis.

### 3.5. Scanning Electron Microscopy Observation

To examine the wax morphology of fruits during storage, SEM observation was referenced to Liu et al. [7] method. Peel disks of 0.5 cm in diameter from the equatorial zone of six fruits were removed by a cork borer and fixed with 2.5% glutaraldehyde solution. These fixed disks were freeze-dried, sputter-coated with gold film in a sputter coater (2 × 10^−4^ MPa, 25 mA, 300 s), and examined by an EVO LS10 SEM (Zeiss International, Jena, Germany). SEM observation was sampled every 10 days.

### 3.6. Effect of Different Cuticle Extracts on Mycelium Growth and Conidial Germination of Penicillium digitatum In Vitro

A bio-assay method according to the procedures of Li et al. [22] with some modifications was used to obtain the effect of cuticle extracts obtained from citrus fruit during storage on the mycelium growth of *Penicillium digitatum.* A volume of 400 μL of different cuticle extractions was sprayed on the surface of Petri dishes filled with PDA and covered loosely for 2 h before inoculation to allow the solvents to evaporate. Chloroform and sterile water were sprayed on the surface of PDA cultures and served as the control. The Oxford cup was placed in the center of each Petri dish and 200 µL of 1 ×10^6^ spores mL^−1^ solution was added. The diameters of the *Penicillium digitatum* mycelia were measured after 7 days at 28 °C. Three replicates were performed for each experiment.

For conidial germination, cuticle extracts were painted onto glass coverslips so that an even film formed after air-drying. Chloroform and sterile water severed as the control. All the coverslips were inoculated with four droplets (2 µL each drop) of 1 × 10^6^ spores mL^−1^ and placed in the lid of an inverted Petri dish so that they were below 0.95 M NaCl-agar. Three coverslips were used per treatment. Petri dishes were then incubated for 24 h in the dark at 28 °C. The number of germinated spores was determined by examination with a light microscope (XSP-30, Phoenix Optical Technology Co., Ltd., Nanchang, China).

### 3.7. Statistical Analysis

The experimental design was completely randomized with three replications. All statistical analyses were performed using Origin 8.0. Data were analyzed by one-way analysis of variance with Duncan’s multiple range tests, and *p* < 0.05 was classified as statistically significant.

## 4. Conclusions

This study reports changes in cuticle chemical composition and morphology during postharvest citrus fruits and reveals their potential role in *Penicillium digitatum* infection. These results show that epicuticular and intracuticular wax content, relative amount of wax classes varied in the different storage stages of citrus fruits. Cutin monomer amounts of fruits presented a decreased trend during storage, while their proportions in the cutin stayed stable. The morphology of the fruit cuticle wax was closely related to the variations in the compositions of the epicuticular wax, and it changed from flattened platelets to small granule-like after storage. The results from in vitro test elucidate the regulatory mode of the chemical composition or morphology of cuticle on infection behavior of *Penicillium digitatum* on ‘Satsuma’ mandarin. The mycelial growth of *Penicillium digitatum* could be promoted by epicuticular wax and conidial germination could be inhibited by cutin at different storage stages.

## Figures and Tables

**Table 1:** Changes in cuticular fraction contents of the ‘Satsuma’ mandarin fruits during storage.

Cuticle Fractions	Cuticular Fraction Contents (μg cm^−2^)				
	Harvest	10 days	20 days	30 days	40 days
Epicuticular wax	1.11 ± 0.18 ^c^	3.22 ± 0.25 ^b^	4.21 ± 0.39 ^a^	3.52 ± 0.63 ^b^	1.35 ± 0.02 ^d^
Intracuticular wax	4.78 ± 0.19 ^b^	3.70 ± 0.14 ^c^	6.09 ± 0.30 ^a^	5.00 ± 0.26 ^b^	3.66 ± 0.55 ^c^
Total wax	5.89 ± 0.21 ^d^	6.92 ± 0.28 ^c^	10.30 ± 0.40 ^a^	8.52 ± 0.74 ^b^	5.01 ± 0.59 ^d^
Cutin	52.50 ± 1.79 ^a^	38.10 ± 0.78 ^b^	39.00 ± 1.16 ^b^	27.80 ± 2.54 ^c^	28.21 ± 5.25 ^c^

Note: Means in the same row with different lower case letters are significantly different (*p* < 0.05).

**Table 2:** Epicuticular wax constituents of ‘Satsuma’ mandarin fruits during storage.

Compositions	Contents of Epicuticular Wax Constituents (μg cm^−2^)				
	Harvest	10 days	20 days	30 days	40 days
Acids	0.73 ± 0.01 ^c^	2.25 ± 0.26 ^b^	3.03 ± 0.11 ^a^	2.63 ± 0.18 ^b^	0.70 ± 0.03 ^c^
Dodecanoic acid	0.03 ± 0.01 ^b^	0.04 ± 0.00 ^b^	0.07 ± 0.01 ^a^	0.11 ± 0.04 ^a^	-
Tetradecanoic acid	0.05 ± 0.01 ^b^	0.10 ± 0.01 ^a^	0.10 ± 0.02 ^a^	-	-
Pentadecanoic acid	0.02 ± 0.01	-	-	-	-
cis-9-Hexadecenoic acid	0.02 ± 0.01 ^b^	0.07 ± 0.03 ^a^	0.11 ± 0.05 ^a^	0.11 ± 0.01 ^a^	-
Hexadecanoic acid	0.40 ± 0.02 ^c^	0.80 ± 0.06 ^b^	1.02 ± 0.13 ^a^	0.80 ± 0.07 ^b^	0.31 ± 0.03 ^c^
9,12-Octadecanoic acid	0.02 ± 0.01 ^b^	0.07 ± 0.03 ^a^	0.11 ± 0.03 ^a^	0.08 ± 0.02 ^a^	-
Oleic acid	0.03 ± 0.00 ^b^	0.16 ± 0.01 ^a^	0.16 ± 0.07 ^a^	0.12 ± 0.04 ^a^	-
Octadecanoic acid	0.12 ± 0.01 ^e^	0.35 ± 0.07 ^c^	0.72 ± 0.08 ^a^	0.49 ± 0.03 ^b^	0.18 ± 0.01 ^d^
Eicosanoic acid	0.04 ± 0.02 ^d^	0.66 ± 0.11 ^b^	0.74 ± 0.13 ^a,b^	0.92 ± 0.19 ^a^	0.21 ± 0.03 ^c^
Alkanes	0.12 ± 0.01 ^d^	0.45 ± 0.05 ^b^	0.68 ± 0.03 ^a^	0.54 ± 0.06 ^b^	0.35 ± 0.02 ^c^
Heptadecane	0.04 ± 0.02	-	-	-	-
Heptacosane	0.02 ± 0.01 ^c^	0.18 ± 0.09 ^a^	0.23 ± 0.05 ^a^	0.22 ± 0.04 ^a^	0.10 ± 0.02 ^b^
Nonacosane	0.02 ± 0.01 ^d^	0.27 ± 0.10 ^b,c^	0.45 ± 0.04 ^a^	0.32 ± 0.04 ^b^	0.25 ± 0.01 ^c^
Hentriacontane	0.04 ± 0.01	-	-	-	-
Terpenoids	0.17 ± 0.01 ^a^	0.12 ± 0.06 ^a,b^	0.20 ± 0.07 ^a^	0.15 ± 0.01 ^a^	0.10 ± 0.01 ^b^
Squalene	0.02 ± 0.00	-	-	-	-
Friedelin	0.15 ± 0.02 ^a^	0.12 ± 0.06 ^a,b^	0.20 ± 0.07 ^a^	0.15 ± 0.01 ^a^	0.10 ± 0.01 ^b^

Note: Means in the same row with different lower case letters are significantly different (*p* < 0.05). “-” presented not detected.

**Table 3:** Intracuticular wax main constituents of ‘Satsuma’ mandarin fruits during storage.

Compositions	Contents of Intracuticular Wax Constituents (μg cm^−2^)				
	Harvest	10 days	20 days	30 days	40 days
Acids	1.71 ± 0.02 ^a^	0.72 ± 0.05 ^d^	1.15 ± 0.07 ^b^	0.83 ± 0.03 ^c^	0.69 ± 0.02 ^d^
Dodecanoic acid	0.03 ± 0.00	-	-	-	-
Tetradecanoic acid	0.13 ± 0.01 ^a^	0.02 ± 0.01 ^b,c^	0.01 ± 0.01 ^c^	0.04 ± 0.01 ^b^	0.03 ± 0.01 ^b^
Pentadecanoic acid	0.07 ± 0.01	-	-	-	-
cis-9-Hexadecenoic acid	0.15 ± 0.01 ^a^	0.01 ± 0.00 ^c^	0.02 ± 0.01 ^b,c^	0.03 ± 0.01 ^b^	0.02 ± 0.01 ^b,c^
Hexadecanoic acid	0.80 ± 0.05 ^a^	0.32 ± 0.02 ^c,d^	0.57 ± 0.03 ^b^	0.38 ± 0.02 ^c^	0.27 ± 0.03 ^d^
Heptadecanoic acid	0.02 ± 0.00	-	-	-	-
9,12-Octadecanoic acid	0.06 ± 0.01 ^b^	0.07 ± 0.02 ^b^	0.11 ± 0.01 ^a^	0.06 ± 0.01 ^b^	0.07 ± 0.02 ^b^
Oleic acid	0.15 ± 0.03 ^a,b^	0.11 ± 0.01 ^b^	0.20 ± 0.03 ^a^	0.07 ± 0.01 ^c^	0.08 ± 0.01 ^c^
Octadecanoic acid	0.25 ± 0.02 ^a^	0.14 ± 0.03 ^b^	0.15 ± 0.04 ^b^	0.19 ± 0.04 ^a,b^	0.13 ± 0.04 ^b^
Tetracosanoic acid	0.05 ± 0.01 ^c^	0.05 ± 0.01 ^c^	0.09 ± 0.01 ^a^	0.06 ± 0.01 ^b,c^	0.09 ± 0.02 ^a,b^
Alkanes	0.45 ± 0.01^e^	0.70 ± 0.06 ^d^	1.27 ± 0.04 ^a^	0.90 ± 0.06 ^c^	1.07 ± 0.06 ^b^
Heptadecane	0.02 ± 0.00 ^b^	0.06 ± 0.01 ^a^	0.03 ± 0.01 ^b^	0.02 ± 0.01 ^b,c^	0.01 ± 0.00 ^c^
Pentacosane	0.04 ± 0.01 ^a^	0.01 ± 0.00 ^b^	0.06 ± 0.01 ^a^	0.05 ± 0.01 ^a^	0.05 ± 0.01 ^a^
Hexacosane	0.03 ± 0.01 ^a^	0.01 ± 0.00 ^b^	0.02 ± 0.01 ^a,b^	0.02 ± 0.01 ^a,b^	0.02 ± 0.01 ^a,b^
Heptacosane	0.14 ± 0.01 ^b^	0.03 ± 0.01 ^b^	0.08 ± 0.02 ^a^	0.04 ± 0.01 ^b^	0.04 ^a^ ± 0.00
Nonacosane	0.16 ± 0.02 ^c^	0.17 ± 0.05 ^b,c^	0.34 ± 0.02 ^a^	0.22 ± 0.02 ^b^	0.30 ± 0.06 ^a,b^
Triacontane	0.02 ± 0.00 ^c^	0.39 ± 0.09 ^b^	0.68 ± 0.04 ^a^	0.50 ± 0.15 ^a,b^	0.60 ± 0.10 ^a^
Hentriacontane	0.04 ± 0.01 ^b^	0.03 ± 0.01 ^b^	0.06 ± 0.01 ^a^	0.05 ± 0.01 ^a,b^	0.05 ± 0.01 ^a,b^
Alcohols	0.27 ± 0.04 ^b^	0.27 ± 0.09 ^b^	0.53 ± 0.05 ^a^	0.52 ± 0.10 ^a^	0.58 ± 0.07 ^a^
13-Docosen-1-ol	0.25 ± 0.05 ^a^	0.07 ± 0.01 ^c^	0.14 ± 0.05 ^b^	0.17 ± 0.07 ^a,b^	0.15 ± 0.04 ^b^
Tetracosan-1-ol	0.02 ± 0.01 ^b^	0.20 ± 0.09 ^b^	0.39 ± 0.09 ^a^	0.35 ± 0.10 ^a,b^	0.43 ± 0.08 ^a^
Terpenoids	2.54 ± 0.11 ^b^	2.06 ± 0.10 ^c^	4.35 ± 0.18 ^a^	2.76 ± 0.15 ^b^	2.27 ± 0.12 ^c^
Farnesol	-	0.01 ± 0.00 ^b^	0.03 ± 0.01 ^a^	-	-
Squalene	0.29 ± 0.09 ^b^	0.12 ± 0.01 ^c^	0.58 ± 0.10 ^a^	0.13 ± 0.09 ^c^	-
Campesterol	0.05 ± 0.01 ^b^	0.05 ± 0.01 ^b^	0.10 ± 0.02 ^a^	0.05 ± 0.01 ^b^	0.06 ± 0.01 ^b^
Stigmasterol	0.04 ± 0.01 ^a,b^	0.02 ± 0.01 ^b^	0.05 ± 0.01 ^a^	0.03 ± 0.01 ^a,b^	0.04 ± 0.01 ^a,b^
Lup-20(29)-en-3-one	0.30 ± 0.07 ^c^	0.35 ± 0.04 ^c^	0.70 ± 0.03 ^a^	0.50 ± 0.10 ^b^	-
Lanosterol	-	0.04 ± 0.01 ^b^	0.10 ± 0.04 ^a^	0.06 ± 0.05 ^a,b^	0.08 ± 0.02 ^a^
Urs-12-en-24-oic acid	-	0.03 ± 0.01 ^b^	0.06 ± 0.01 ^a^	0.04 ± 0.01 ^a,b^	0.05 ± 0.01 ^a,b^
β-Amyrin	0.36 ± 0.06 ^c^	0.35 ± 0.05 ^c^	0.71 ± 0.08 ^a^	0.48 ± 0.11 ^b,c^	0.52 ± 0.08 ^b^
Friedelin	1.50 ± 0.12 ^b^	1.09 ± 0.09 ^c^	2.02 ± 0.16 ^a^	1.47 ± 0.14 ^b^	1.52 ± 0.13 ^b^

Note: Means in the same row with different lower case letters are significantly different (*p* < 0.05); “-” presented not detected.

**Table 4:** Cutin monomer compositions of the ‘Satsuma’ mandarin fruits during storage.

Compositions	Contents of Cutin Monomer Compositions (μg cm^−2^)				
	Harvest	10 days	20 days	30 days	40 days
Cinnamic acid	13.91 ± 0.82 ^a^	10.29 ± 0.73 ^b^	8.58 ± 0.32 ^c^	6.67 ± 0.38 ^d^	6.26 ± 0.21 ^d^
Pentadecanoic acid	2.21 ± 0.07 ^a^	1.64 ± 0.12 ^b^	1.76 ± 0.09 ^b^	1.08 ± 0.10 ^c^	1.04 ± 0.05 ^c^
Hexadecanoic acid	4.10 ± 0.13 ^a^	3.31 ± 0.28 ^c^	3.71 ± 0.17 ^b^	3.92 ± 0.09 ^a,b^	3.95 ± 0.23 ^a,b^
Hexadecanedioic acid	2.20 ± 0.06 ^b^	1.91 ± 0.05 ^c^	2.73 ± 0.07 ^a^	1.81 ± 0.05 ^c^	1.69 ± 0.02 ^d^
Octadecanoic acid	17.06 ± 0.52 ^a^	12.95 ± 0.24 ^c^	14.04 ± 0.34 ^b^	9.17 ± 0.12 ^d^	9.33 ± 0.29 ^d^
Octadecanedioic acid	2.89 ± 0.10 ^a^	1.90 ± 0.08 ^c^	2.73 ± 0.06 ^a^	2.09 ± 0.03 ^b^	1.41 ± 0.01 ^d^
Tetracosanoic acid	6.72 ± 0.32 ^a^	2.67 ± 0.12 ^d^	3.90 ± 0.21 ^b^	2.50 ± 0.13 ^d^	3.10 ± 0.09 ^c^
Octacosanoic acid	1.31 ± 0.02 ^a^	1.14 ± 0.07 ^b^	0.78 ± 0.03 ^c^	-	-
Phenol	2.10 ± 0.16 ^a^	2.29 ± 0.13 ^a^	0.78 ± 0.06 ^c^	0.56 ± 0.02 ^c^	1.41 ± 0.05 ^b^

Note: Means in the same row with different lower case letters are significantly different (*p* < 0.05); “-” presented not detected.

## Abbreviations

1-MCP	1-methylcyclopropene
BSTFA	bis-*N*,*N*-(trimethylsilyl)-trifluoroacetamide
EGCG	epigallocatechin-3-gallate
GC-MS	gas chromatography-mass spectrometry
ROS	reactive oxygen species
